# Boosting Tadalafil Bioavailability via Sono-Assisted Nano-Emulsion-Based Oral Jellies: Box–Behnken Optimization and Assessment

**DOI:** 10.3390/pharmaceutics14122592

**Published:** 2022-11-24

**Authors:** Hany S. M. Ali, Sameh A. Ahmed, Abdulmalik A. Alqurshi, Ali M. Alalawi, Ahmed M. Shehata, Yaser M. Alahmadi

**Affiliations:** 1 Department of Pharmaceutics and Pharmaceutical Technology, College of Pharmacy, Taibah University, Al-Madinah Al-Munawarah P.O. Box 344, Saudi Arabia; 2 Department of Pharmaceutics, Faculty of Pharmacy, Assiut University, Assiut 71526, Egypt; 3 Department of Pharmacognosy and Pharmaceutical Chemistry, College of Pharmacy, Taibah University, Al-Madinah Al-Munawarah P.O. Box 344, Saudi Arabia; 4 Department of Pharmaceutical Analytical Chemistry, Faculty of Pharmacy, Assiut University, Assiut 71526, Egypt; 5 Department of Pharmacology and Toxicology, College of Pharmacy, Taibah University, Al-Madinah Al-Munawarah P.O. Box 344, Saudi Arabia; 6 Department of Pharmacology and Toxicology, Faculty of Pharmacy, Beni-Suef University, Beni-Suef 62521, Egypt; 7 Department of Clinical and Hospital Pharmacy, College of Pharmacy, Taibah University, Al-Madinah Al-Munawarah P.O. Box 344, Saudi Arabia

**Keywords:** tadalafil, nano-emulsion, ultrasonic processing, Box–Behnken design, jelly, bioavailability

## Abstract

Tadalafil (TAD) is a poorly soluble, phosphodiesterase inhibitor used to treat erectile dysfunction. The primary goal of this project was to prepare nano-emulsions using ultrasonic technology to address TAD bioavailability concerns. The Box–Behnken design was employed to find prominent correlations between factors impacting the sono-emulsification process. The emulsifier concentration, amplitude level, and ultrasonication time were the independent factors, whereas the average droplet size (ADS) and polydispersity index (PDI) were designated as the response variables. TAD-loaded nano-emulsions (93–289 nm) were generated and the emulsifier concentration showed a crucial role in directing emulsion droplet size. The model desirability function was utilized to optimize a nano-emulsion with a small ADS (99.67 ± 7.55 nm) and PDI (0.45 ± 0.04) by adjusting the emulsifiers concentration, amplitude level, and ultrasonication time at 9.85%, 33%, 49 s, respectively. The optimized nano-emulsions did not demonstrate any precipitation or phase separation after stability stress tests. TAD jellies were formulated based on the optimized nano-emulsion and subjected to in vitro evaluation for physical characteristics; TAD content, pH, spreadability, viscosity, syneresis, and taste-masking ability. An optimized nano-emulsion-based jelly (NEJ) formulation showed more than 96% drug dissolution in 30 min relative to 14% for the unprocessed TAD. In vivo assessment of NEJ in experimental rats demonstrated a significant enhancement (*p* < 0.05) of TAD bioavailability with an AUC_0–24h_ of 2045 ± 70.2 vs. 259.9 ± 17.7 ng·h·mL^−1^ for the unprocessed TAD. Storage stability results revealed that NEJ remained stable with unremarkable changes in properties for 3 months. Overall, NEJ can be regarded as a successful therapeutic option for TAD administration with immediate-release properties and improved bioavailability.

## 1. Introduction

Tadalafil (TAD) is a phosphodiesterase-5 (PDE5) inhibitor officially approved to treat erectile dysfunction (ED) [1]. TAD is deemed the most potent and most selective among the PDE5 inhibitors [2]. Contrary to sildenafil and vardenafil, TAD retains a lower inhibitory impact on PDE6, with a lower incidence of visual adverse effects, <0.1% [3]. Accordingly, TAD is clinically agreed to treat erectile dysfunction including challenging cases [4]. The Biopharmaceutics Classification System (BCS) categorizes TAD as a class II drug [5]. In spite of its high permeability, TAD’s bioavailability is restricted by its low solubility and dissolution rate, resulting in unpredictable absorption, fluctuating medication blood levels, and inconsistent therapeutic effects [6].

Nano-emulsions (NE) can be defined as a biphasic dispersion of two immiscible liquids, e.g., oil and water, either water in oil (W/O) or oil in water (O/W) droplets stabilized by an interfacial film of amphiphilic surfactant [7]. The average droplet diameter in NE substantially varies across different studies. Although some studies were shown to expand the maximum size value of the dispersed phase to 500 nm [8] or even to 1000 nm [9], there is a wide agreement that the size of the nano-emulsified droplet should be less than 200 nm [7,10,11,12]. Features of NE such as stability, viscosity, appearance, color, texture, and shelf life are all affected by droplet size and polydispersity of the NE [13]. In pharmaceutical research, NEs are used to increase cell absorption and bioavailability of several poorly soluble medications by improving their solubility and dissolution, modulating drug release, and minimizing systemic adverse effects by targeting particular disease sites [14,15,16]. Regarding tadalafil, NEs were found effective in improving solubility and dissolution rate because of the distinct surface area increment resulting from the nanosized emulsion droplets [5,17,18,19]. Recently, the interest in employing ultrasound (acoustic or sono-processing) for nano-emulsification has increased. Ultrasound devices produce disrupting forces that are able to convert immiscible liquids into small, stable droplets using high-intensity ultrasound waves that are non-toxic, safe, and environmentally friendly [20,21]. Ultrasound devices create acoustic cavitations, which collapse air bubbles in the system [22,23]. When compared to microfluidizers, ultrasound is considered more feasible for scale-up manufacturing; additionally, it is more practical for maintenance and aseptic processing. Several studies have demonstrated that, in emulsification processes, ultrasound has emerged as a superior instrument to the rotor-stator in terms of achieving smaller droplet size and great energy efficiency [24]. Furthermore, several studies have demonstrated that the amount of surfactant required to produce an emulsion with the desired droplet diameter may be dramatically lowered by introducing the low-energy-consuming ultrasound method [23]. Additionally, acoustically produced emulsions were found to be particularly stable and homogeneous when compared to mechanical approaches [16,25].

Jellies, translucent semi-solid formulations, are pharmaceutically utilized for lubrication and therapeutic purposes [26]. Orally ingested medicated jellies are regarded as an effective option for a wide range of patient groups, particularly youngsters and dysphagic patients; additionally, they were included as a type of dosage form in the Japanese Pharmacopoeia, 16th Edition [27]. Several pieces of research were conducted on jellies in order to address bioavailability issues and enhance patient compliance [26]. The orally ingested jellies are also easily applicable to the older population, who require ED therapy and may have difficulty in swallowing tablets.

In light of this, the current study was performed to improve the bioavailability of TAD using sono-assisted NEs. The Box–Behnken Design (BBD) was employed to identify the impact of different sono-operating conditions. BBD is a response surface methodology (RSM) design that involves fewer runs in a three-factor experimental design [28]. In the pharmaceutical field, the majority of NE optimization studies have focused on self-nano-emulsified drug delivery systems (SNEDDS), and only a few studies discussed the optimization of drug-loaded sono-processed NEs using RSM. Moreover, the size ranges of generated droplets were mostly above 200 nm [29,30]. Here, the generated BBD model was utilized to optimize TAD-loaded NEs with a minimized droplet size and polydispersity index. The optimized NEs were then formulated as orally ingested jellies and subjected to in vitro characterization. The aim of the project was extended to assess the bioavailability of TAD when released from the developed jelly formulation.

## 2. Materials and Methodology

Tadalafil (98%) was kindly obtained from SAJA Pharmaceuticals, (Jeddah, KSA). Cinnamon essential oil (CEO) was bought from Now foods (Bloomingdale, IL, USA). Propylene glycol mono caprylate (Capryol^TM^ 90) was purchased from UFC Biotechnology (Amherst, NY, USA). PEG 40 hydrogenated castor oil (Cremophor^®^ RH 40) and sodium carboxy methylcellulose (Sodium CMC, average molecular weight ~250,000, degree of substitution 0.7) were bought from Sigma-Aldrich Chemie-GmbH (Steinheim, Germany). Diethylene glycol monoethyl ether (Transcutol^®^ HP, TR) was gifted from Gattefossé (Cedex, France). A Millipore water filtration system (Bedford, MA, USA) was used to obtain ultra-pure water. Sildenafil (SLD, 97.5%) was purchased from Acros Organics (Geel, Belgium).

### 2.1. Preformulation

#### 2.1.1. Solubility

Solubility of TAD was assessed in the tested emulsion components, CEO, Capryol^TM^ 90, CR and TR according to published procedures [31]. In brief, TAD was introduced in excess of 2 g of each liquid to closed glass vials and shaken (Ika^®^ KS 260 B, Staufen, Germany) at 25 ± 0.5 °C for 48 h at a 100 rpm shaking rate. The vial contents were then centrifuged for 15 min at 10,000 rpm (Centrifuge Z 206 A;HermleLabortechnik GmbH, Wehingen, Germany). A 0.45 mm-syringe filter was used to filter the supernatant and a volume (0.1 mL) was taken and properly diluted with methanol. Analysis for TAD content was carried out according to the validated high-pressure liquid chromatography, HPLC, method [32]. The HPLC system involved a PU 2080 pump (Jasco Corporation, Tokyo, Japan) with a UV/VIS detector (UV 2075 plus) (Jasco Corporation, Tokyo, Japan) covering the range of 200–400 nm. A reverse phase C18 column (250 × 4.6 mm, 5 μm, HiQ Sil, Tokyo, KyaTech Corporation, Japan). TAD separation mobile phase was composed of phosphate buffer pH 3.2 and acetonitrile (50:50 *v*/*v*). The mobile phase was introduced at a constant flow rate of 1.0 mL/min and the detector was adjusted at 295 nm. Results of three tested samples from each experiment was displayed as the mean ± standard deviations (SD).

#### 2.1.2. Determination of the Hydrophile–Lipophile Balance (HLB)

The HLB of the emulsifier system was determined to find the proper ratio of components required to emulsify the oily phase. For determination of the HLB of the oily phase (CEO + Capryol 90), a series of oil/water emulsions was prepared by keeping the oil phase, S_mix_ and water at 7%, 10% and 83%, respectively. The tested ratio of CR:TR were 1:1, 2:1, 2.5:1 and 3:1 (*w*/*w*). The reported HLB of CR and TR were 15 and 4.2, respectively [33]. The total HLB value of mixed surfactants (S_mix_) can be calculated according to Equation (1):

HLB mix = fa. HLB_a_ + fb. HLB_b_ (1)

where HLB_a_ represented surfactant HLB values and HLB_b_ represented cosurfactant HLB values. The weight fractions of surfactant and cosurfactant are denoted by fa and fb, respectively [34]. Formulations of 10 g were prepared for the HLB test. The lipophilic phase was gently introduced to water with stirring at 3000 rpm for 5 min at 25 ± 0.5 °C using a stirring plate (IKA, model C-MAG HS 7). The optimal HLB value necessary to stabilize the emulsions was characterized macroscopically and by the percentage transparency (% transparency) after 24 h. Macroscopic characteristics (creaming, coalescence, and/or the existence of phase separation) were assessed by visual examination [35]. Transparency (%) of the produced mixtures was determined using a UV6100 PC spectrophotometer (EMC lab, Duisburg, Germany) at wavelength of 650 nm using deionized water as the blank.

### 2.2. Box–Behnken Design, BBD

The prepared NEs were analyzed by a 3-factor 3-level Box–Behnken design (BBD) using Minitab^®^ 17 (Minitab Inc., State College, PA, USA). The surfactant concentration, (% S_mix_, X_1_; 5–15%), amplitude level (%, X_2_; 20–40%), and sonication time (X_3_; 30–90 s) were designated as the independent variables. These variables were altered at three levels: low (coded as −1), middle (coded as 0), and high (coded as +1). The BBD requires 15 experimental runs (Table 1 and Table 2) with three replicated center points (X_1_ = 10%, X_2_ = 30%, and X_3_ = 60 s) for a more consistent estimate of prediction variance over the whole design space. The selected responses or dependent variables were average droplet size, ADS, (Y_1_) and polydispersity index, PDI, (Y_2_). The target of BBD is to minimize Y_1_ and Y_2_. 

The obtained responses were fitted separately to a non-linear, full quadratic, Equation (2):

(2) $$Y_{i}=b_{0}+b_{1}X_{1}+b_{2}X_{2}+b_{3}{\;X}_{3}+b_{11}X_{1}^{2}\;+b_{22}{\;X}_{2}^{2}+b_{33}X_{3}^{2}+b_{12}{\;X}_{1}X_{2}+b_{13}{\;X}_{1}X_{3}\;+b_{23}{\;X}_{2}X_{3}$$

where Y_i_ is the response associated with each variable (factor); b_0_ denotes the intercept; b_1_–b_33_ denote the regression coefficients acquired from the experimental values; and X_1_, X_2_, and X_3_ are the codes of independent variables levels chosen from the preparatory research. The interaction terms (X_12_, X_13_, and X_23_) indicate how the answer changes when two factors are altered at the same time [36].

The model’s significance was determined using ANOVA, the lack of fit test, and the multiple correlation coefficient (R^2^) test. For a significant fit of the model to the quadratic equation, the model *p*-value should be less than 0.05. Data variation around the fitted value is assessed by the lack of fit test and it should be not significant (*p*-value > 0.05) in comparison to the pure error. The R^2^ value reflects the degree of variance around the mean (multiple correlation coefficient test) and it should be close to one. In addition, the numerical optimization process by means of the desirability function was employed to choose the optimum NE [37,38,39]. 

### 2.3. Ultrasonic Set Up

Sono-assisted emulsification was achieved using a 24 KHz A Vibra-Cell^TM^ Ultrasonic Liquid Processor (Sonics & Materials, Inc., Newtown, CT, USA). This setup includes a power generator, a sealed converter, and a horn microtip [25]. The generator transforms 60 Hz alternating power to 24 KHz, whereas the converter changes the generated energy into mechanical vibrations. The converter contains an internal transducer made of piezoelectric lead zirconate titanate crystals (PZT) that could expand and contract when subjected to alternating currents. The converter vibrates and transmits this motion to a 22 mm diameter horn tip that was immersed in the coarse emulsion. With adjustable amplitude control, a maximum power output of 750 W may be provided. The magnitude of amplitude appears as a % of the maximum amplitude. Nevertheless, the maximum permitted operating amplitude for the horn microtip system was 40% due to the increased intensity of cavitation induced by the more concentrated ultrasonic field compared to the conventional horn tip. In this study, the ultrasonic processing variables included the amplitude level (%) and duration time (s). Processing via a pulsed mode with 15 s ON followed by 10 s OFF was activated during sonication time, as it is more efficient to produce NEs with significantly smaller droplet size and PDI when compared with the continuous mode [40]. 

### 2.4. Nano-Emulsification

Fifteen NE preparations were produced incorporating TAD at a loading level of 2.5 mg/g. TAD was added to a specified quantity of the oily phase (0.3% CEO + 6.7% Capryol^TM^ 90) and sonicated till complete dissolution. Different amounts (5–15%, *w*/*w*) of S_mix_ (CR:TR, 2.5:1, *w*/*w*) were added to the oily phase. The constituents were mixed by a vortex shaker and then warmed to 40 °C in a water bath to obtain a clear homogenous mix. The aqueous phase was also warmed to the same temperature before being added to the oily phase and pre-emulsified via a digital homogenizer (Ultra-Turrax^®^ T 25; IKA, Königswinter, Germany) at 4000 rpm for 5 min to form a coarse emulsion. The coarse emulsion was sono-processed as described in Section 2.3. An ice bath was used to minimize temperature increase throughout the ultrasonication progression [40]. The influences of % amplitude level, ultrasonication time (min), along with the % emulsifiers system were studied. 

#### 2.4.1. Determination of Droplet Size and Polydispersity Index

The average droplet size (ADS) and polydispersity index (PDI) of the prepared NEs were determined by Microtrac S3500 (Microtrac Inc., Montgomeryville, PA, USA). Proper dilution with double distilled water was carried out before measurement to prevent the influence of multiple scattering. NEs were measured three times for 120 s at 25 °C [41,42].

#### 2.4.2. Nano-Emulsion Stability

The optimized TAD nano-emulsion (OTN) was subjected to six cycles between refrigeration at 4 °C and heating to 45 °C with storing for 48 h. OTN was further centrifuged at 3500 rpm for 30 min. Finally, OTN was subjected to freeze–thaw cycles (*n* = 3) at −21 °C and 25 °C, with storage at each temperature for 48 h [43,44]. After each test of stability evaluation, samples of OTN were checked for instability signs such as phase separation or precipitation and also tested for ADS and PDI, as described in Section 2.4.1 [39].

#### 2.4.3. Robustness to Dilution

Different dissolution media, 0.1N HCl and phosphate buffers (pH 6.8) were used to dilute OTN to 10, 100, and 1000 times. Samples after dilutions were evaluated for ADS, PDI and any instability features after storing for 24 h [45].

#### 2.4.4. Zeta Potential

Determination of Zeta potential of OTN was performed by Microtrac S3500 (Microtrac Inc., Montgomeryville, PA, USA). OTN was diluted 10 times prior measurement [37].

### 2.5. Tadalafil Jellies

TAD-loaded nano-emulsion-based jellies (TAD-NEJ) were developed by adding sodium carboxymethyl cellulose (Sodium CMC) as the gelling agent at 0.5–3.5% *w*/*w* concentrations. Sodium CMC to was allowed to swell in distilled water for 24 h. The OTN was slowly added to the viscous solution of gelling agents with mechanical stirring (Ultra-Turrax^®^ T 25; IKA, Königswinter, Germany). The final concentration of TAD obtained in gels was 0.5 mg/g. Stevia (1% *w*/*w*) was chosen as the sweetener in NEJ formulations because it is a natural sweetener and successfully used as an alternative to sucralose for diabetic users without affecting blood glucose levels, hemoglobin A1c, insulin, and lipid levels [46]. Due to the bitter taste of TAD, appropriate flavoring and coloring agents were also included in NEJs’composition. In order to enhance users’ compliance, Strawberry flavoring agent (0.1% *w*/*v*) together with amaranth color (0.1% *w*/*v*) were included in the formulations [47]. Methyl paraben (0.2%, *w*/*v*) was incorporated to preserve the formulations Details of NEJ formulations are presented in Table 3.

### 2.6. Evaluation of Tadalafil Jellies

#### 2.6.1. Physical Characteristics

Consistency of the NEJs was evaluated by physical observation, while texture was assessed by mildly rubbing the jelly between two fingers in terms of stickiness and grittiness. [26]. Transparency of NEJs was assessed on a scale of one to five. Jellies were placed in clear test tubes and observed against a white and black background. The clear translucent jelly received a score of five, while a score of one was assigned to the jelly with minimum transparency [48].

#### 2.6.2. Drug Content

Jelly amounts equivalent 2.5 mg of TAD were introduced in 100 mL volumetric flasks and dissolved in appropriate volumes of methanol with vigorous shaking for 5–10 min. TAD contents were quantified using HPLC procedures as previously explained (Section 2.1.1).

#### 2.6.3. Viscosity

Brookfield^®^ viscometer (Model: DV II+, Brookfield Engineering, Middleborough, MA, USA) was used to determine viscosity of NEJ formulations. The spindle no. LV-4 (64) was used at the rotation of 10 rpm. Measurements were performed in triplicates at room temperature (25 ± 5 °C) [48,49].

#### 2.6.4. pH

The pH of the developed NEJ was measured by a digital pH meter. Amounts of 0.5 g of formulations were dissolved in 50 mL of distilled water for the preparation of 1% *w*/*v* solution. The pH meter’s electrode was dripped in the resultant solution and allowed to equilibrate for 60 s before recording the treading [47].

#### 2.6.5. Syneresis

Syneresis is serious challenge associated with oral jellies. Syneresis can be defined as the contraction of jelly upon standing and separation of water from the jelly. The formulated NEJs were examined for marks of syneresis at room temperature (25 ± 5 °C) and 8 ± 1 °C [50].

#### 2.6.6. Taste Masking

Assessment of the taste-masking characteristics for TAD-NEJs involved a double-blind crossover investigation evaluating gustatory reactions [51]. Six healthy adult male human volunteers aged 26–59 years and weighing 65–90 kg participated in the study. The study procedure followed the World Medical Association’s ethical principles for medical experiments involving human participants (the Helsinki Declaration). Approval of the research protocol was obtained from the Ethics Committee at the College of Pharmacy, Taibah University before beginning this study(Institutional Protocol Reference: COPTU-REC-35-20220621, approval date: 21 June 2022). Proper consent was taken from the volunteers after describing the research topic in vernacular to them. During taste assessment, volunteers were requested to maintain a normal dietary condition (avoiding high-calorie and junk foods), definite water intake and normal physical activity. Every subject was given a dosage of the jelly equal 2.5 mg of TAD to put in their mouth for 10 s and then spit out. A bitterness intensity scale involving five levels was used; 0 = tasteless, 1 = tolerable, 2 = minor, 3 = moderate, and 4 = intense [49,52].

#### 2.6.7. Differential Scanning Calorimetry

Calorimetric analysis was carried out for the unprocessed TAD, the optimized NEJ formulation and the corresponding blank (TAD free) formulation using DSC 60 (Shimadzu Corporation, Kyoto, Japan). In brief, accurately weighted samples were introduced in aluminum pans and crimp-sealed. In the DSC chamber, samples were allowed to equilibrate at 25 °C and then subjected to heating runs over a temperature range of 25–350 °C at a heating rate of 5 °C/min. [53].

#### 2.6.8. Reconstitution

A sample of the optimized NEJ formulation (100 mg) was vortexed for 30 s in 10 mL of distilled water. The resultant NE was incubated for 30 min at room temperature before the supernatant was removed for ADS and PDI measurement as mentioned previously [54].

#### 2.6.9. Dissolution

Dissolution of the optimized NEJ formulation was carried out using USP type 2 (Paddle) apparatus in 1000 mL 0.1 N HCl kept at 37 ± 0.5 °C and RPM equal to 100 [6,55]. Unprocessed TAD (UNT) and a commercial tablet formulation containing 2.5 mg TAD (CFT) were also evaluated for their dissolution profiles using the same method to obtain comparative data. Samples of 5 mL were withdrawn from the dissolution medium (n = 3) periodically and amount of released TAD was estimated by the previously explained HPLC procedures (Section 2.1.1).

#### 2.6.10. Stability

Samples of the optimized jelly formulation were kept in a desiccator at room temperature (25 ± 5 °C) and 60% relative humidity (RH) for 3 months [56]. The optimized jelly was tested after 3 months for changes in drug content, viscosity, consistency, ADS, PDI and %Q_10min_. The stability was assessed by comparing the initial results to the post-storage results (Zeb et al. 2016).

#### 2.6.11. Bioavailability

##### Animals and Sampling

The in vivo research was conducted in the animal house at the College of Pharmacy, Taibah University. Furthermore, all operations followed the US government’s rules for the use and care of vertebrate animals in testing, research, and training. The Ethics Committee at the College of Pharmacy, Taibah University approved the in vivo study protocols prior to beginning this study (Approval number: COPTU-REC-17-20210705). The experimental animals were divided into three groups of six healthy male Sprague Dawley rats (250 ± 20 g). Rats were kept for 5 days in the animal house prior to testing to allow them to adapt. Rats were given a standard rat diet and had free access to tap water while housed in a controlled setting (22 ± 3 °C, 50 ± 5% relative humidity), and a 12 h light/dark cycle). Rats were starved for an overnight period before tests. To ensure accurate dosage administration, NEJ were dispersed in suitable quantities of deionized water and delivered to rats at doses of 1 mg/Kg through oral gavage with blunt intragastric tubing; this was followed by 0.5 mL of deionized water for cleaning purposes. The commercial tablet formulation (CFT) and unprocessed TAD (UNT) were administered to groups II and III of tested animals, respectively.

##### Tadalafil Determination

In a 1.5 mL Eppendorf tube, a 100 µL aliquot of plasma was deposited and then mixed with 10 µL of the internal standard solution (SLD; 500 ng/mL) and 200 µL of methanol. The tube contents were subjected to vortex mixing for 20 S and centrifugation at 15,000 rpm for 5 min at 25 °C. A validated ultraperformance liquid chromatography method with electrospray ionization and tandem mass detection (UPLC-ESI-MS/MS, Agilent, CA, USA) was employed to determine TAD levels in the processed samples [57]. Quantification was achieved using positive electrospray ionization (ESI) in the MRM mode with the transition’s m/z 390.2 → 268 for TAD and 475.2 → 283 for SLD. The dwell time was set to 25 milliseconds for each MRM transition. The following were the ESI Jet Stream source parameters: gas temperature, 300 °C; gas flow, 10 L/min; nebulizer gas, 15 psi; capillary voltage, 4000 V; fragmentor voltage, 135 V for TAD and 170 V for SLD (IS); collision energy (CE), 10 V for TAD and 45 V for SLD (IS); capillary voltage, 4000 V; fragmentor voltage, 135 V for TAD and 170 V (IS). The chromatographic separation was performed on an Agilent Eclipse Plus C18 RRHD (50 2.1 mm, 1.8 m) column in a reversed-phase mode. The mobile phase (5.0 mM ammonium acetate with 0.1 percent formic acid and acetonitrile with 0.1 percent formic acid (55:45, *v*/*v*) was delivered at a flow rate of 0.4 mL min^−1^. The data were processed using Mass Hunter Quantitative Data Analysis software (Agilent Technologies, Santa Clara, CA, USA).

##### Pharmacokinetic Parameters

The highest reading of plasma drug concentration (C_max_) and its corresponding time (t_max_) was obtained from plasma concentration curves. The area under the curves from zero to the last examined time and infinity (AUC_0–24h_ and AUC_0–∞_) was calculated by the linear trapezoidal method. Equation (3) was used to calculate the % relative bioavailability [58].

(3) $$\%\;\mathrm{Relative}\;\mathrm{Bioavailability}=\frac{{\mathrm{AUC}}_{0–\infty }\;\mathrm{of}\;\mathrm{NEJ}\;\mathrm{formulation}}{{\mathrm{AUC}}_{0–\infty }\;\mathrm{of}\;\mathrm{commercial}\;\mathrm{formulation}}$$

### 2.7. Statistical Analysis

The obtained results were proceeded by the Minitab 17^®^ statistical software (Minitab Inc., State College, PA, USA). One-way analysis of variance (ANOVA) and the Student’s *t*-test were used to analyze the obtained data. *p*-values less than 0.05 were judged as a statistically significant findings.

## 3. Results and Discussion

### 3.1. Preformulation

A blend consisting of cinnamon essential oil (CEO) and Capryol^TM^ 90 was used as the oily phase to prepare the NEs. The surfactant, Cremophor^®^ RH 40 (CR) and co-surfactant, transcutol^®^ HP (TR) were selected based on the miscibility with the oily phase (data not shown). TAD demonstrated a very low aqueous solubility (0.01 ± 0.009) mg·g^−1^. The low solubility value is considered the main restricting factor for in vivo absorption of TAD [59]. However, the solubility values of TAD were 54 ± 3.2, 9.12 ± 1.7, 26.60 ± 3.1 and 29.60 ± 2.7 mg·g^−1^ in CEO, Capryol 90, CR and TR, respectively (Table 4). In the literature, the ability of the CEO to dissolve some drug entities made it suitable for use as the oil component in pharmaceutical NEs. Reported examples included rosuvastatin calcium [60] and candesartan cilexetil [43]. Higher drug solubility in the oil helps stabilize formulations as fewer amounts of the oil are needed for TAD solubilization, minimizing any undesirable actions of the essential oil [61]. In addition to its good solvent capability, components of CEO are able to stimulate the relaxation of human and rat corpus cavernosum and enhance erectile ability [62]. Capryol 90 oil with medium-chain length and amphiphilic properties was used frequently in NE formulations [45,63,64]. In this research, Capryol 90 showed a reasonable solubilization potential toward TAD and was added to minimize the amount required of the CEO. The selection of the surfactant based on maximum drug solubility is controversial since the surfactant with the highest drug solubility does not necessarily demonstrate the needed oil emulsification [44]. Thus, the choice of surfactant or cosurfactant in this investigation was based mostly on emulsification efficiency rather than TAD solubility. However, it would have been an added benefit when the selected surfactant and cosurfactant demonstrated reasonable drug solubilization as well [5]. Nonionic surfactants, which are purportedly less harmful and irritating than other forms of surfactants, are gaining popularity in pharmaceutical NEs [65]. Nonionic surfactants are also recognized to be less impacted by pH and ionic strength variations [44]. A critical consideration in selecting surfactants is the appropriateness of their HLB value. The HLB value considers the contributions of hydrophilic and lipophilic components of the surfactant molecule [66]. In reality, oils would necessitate different HLB values with mutual surfactant/cosurfactant chemical compatibilities [67]. According to the literature, surfactants with HLB values of more than 10 often stabilize O/W NEs. [68]. Some reported studies showed that proper combinations of low (HLB of four to six) and high (HLB of more than 10) surfactants were beneficial to develop a stable NE [5]. According to the HLB study, the combination of CR and TR in 2.5:1 (*w*/*w*) produced a homogeneous emulsion with a % transparency of 97.5. The calculated HLB value for this combination, according to Equation (1), was 11.922.

### 3.2. Box–Behnken Design (BBD)

**Figure 1:**
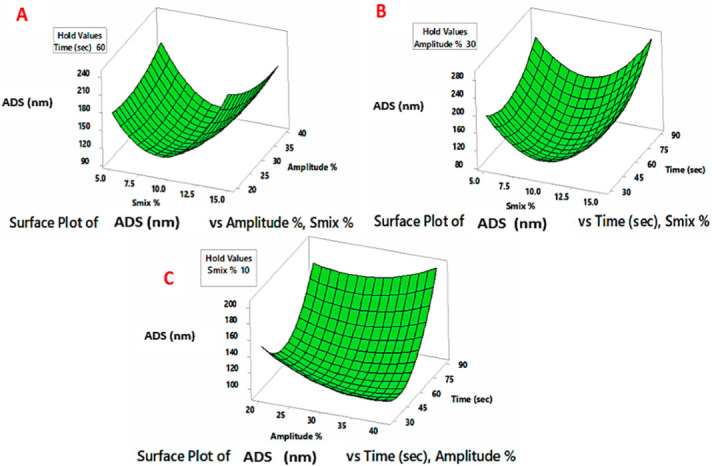
Diagrams of surface response demonstrating the impact of processing parameters on average droplet size (ADS). (**A**) Impact of % S_mix_ and amplitude % on ADS; (**B**) Impact of % S_mix_ and sonication time on ADS; (**C**) Impact of time of % amplitude and sonication time on ADS.

**Figure 2:**
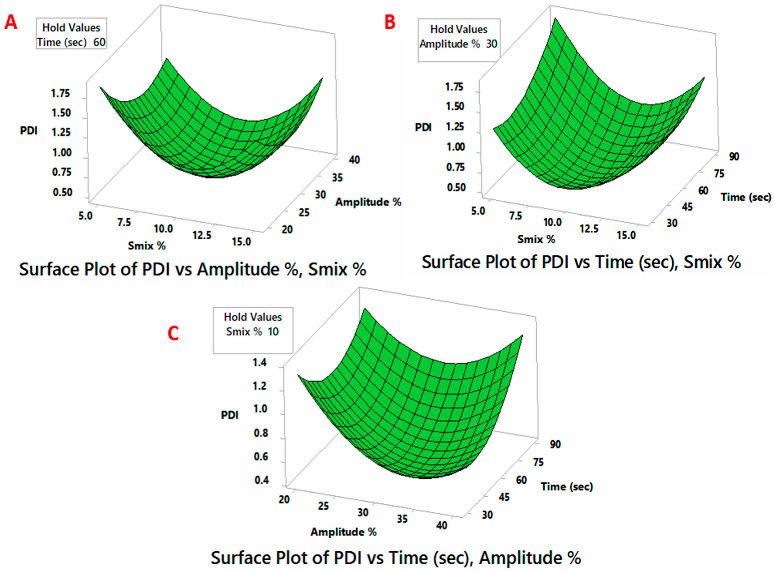
Diagrams of surface response demonstrating the impact of processing parameters on polydispersity index (PDI). (**A**) Impact of % S_mix_ and amplitude % on PDI; (**B**) Impact of % S_mix_ and sonication time on PDI; (**C**) Impact of time of % amplitude and sonication time on PDI.

A three-factor, three-level BBD was utilized to analyze the variation in ADS and PDI of the NEs as a consequence of preparation variables (% S_mix_, % amplitude, and sonication time). The BBD matrix includes fifteen points that reflect the various combinations of the independent factors and the responses of droplet size and PDI values, as observed in Table 2. The range of ADS (Y_1_) for the produced NEs was 93 ± 9.8 to 289 ± 30.6 nm, whereas, the range for PDI values (Y_2_) was 0.48 ± 0.06 to 1.91 ± 0.08. The experimental data were utilized for generating the quadratic polynomial model coefficients, whereas surface graphs were created for each dependent variable to illustrate the response functions (Figure 1 and Figure 2). The adequacy of the BBD models was verified by ANOVA, lack of fit and multiple correlation coefficient (R^2^) tests provided by the Minitab software (Table 5). The *p*-value (<0.05) of the coefficient indicated their significant effect on the obtained responses [41]. The lack of fit test is an additional, useful statistical measure for determining the model’s fitness. It compares residual error to pure error from duplicated design points (three center points in the current study). The lack of fit test should be insignificant (*p*-value > 0.05) relative to the pure error, hence, a model with a non-significant lack-of-fit value is highly desirable for the prediction efficiency [69]. In the present study, the corresponding model’s F-values of 240.44 and 126.15 for Y_1_ and Y_2_ imply significant models. The coefficients of determination (R^2^) for the ADS model (0.9977) and PDI (0.9956) suggest that the model has a high degree of fitness as it approaches the unit [70]. The positive sign of the coefficient implies a collaborative impact, whereas the negative sign shows the opposite impact of such a variable on the response. Moreover, greater coefficient values imply a greater influence on the responses. [36].

#### 3.2.1. Average Droplet Size (ADS)

In NE assessment, emulsion droplet size is crucial as it governs the sequential processes of drug release and absorption. The interfacial surface area available to absorb drugs will be greater for very small-sized droplets. In ultrasound-assisted nano-emulsification, precise energy input is required to initiate cavitation, described by bubble creation, expansion, and implosive collapse in a liquid medium [23]. The final droplet size is controlled by the consequence of droplet break-up and coalescence. Break-up of internal droplets is principally governed by cavitation-induced shear forces transmitted to droplets along with droplet resistance to deformation, whereas droplet coalescence is controlled by the emulsifier’s capability to be adsorbed onto the newly generated interface, which is largely dependent on the surface activity and concentration of the emulsifier [29,71]. Incorporation of proper emulsifiers at suitable concentrations is crucial for the production of nano-emulsions because they reduce the interfacial tension, and hence the stress necessary for droplet deformation. The non-ionic surfactant CR was chosen as the emulsifier in this study owing to its low toxicity and enhanced emulsifying performance with a higher HLB number, 15. CR is a recognized *p*-gp and CYP3A inhibitor and is used in the commercially available micro-emulsion formulation of cyclosporin, Neoral^TM^ [65,72]. The selected cosurfactant, Transcutol HP, TR, was successfully used in combination with CR to generate nano-emulsions because of its low viscosity in addition to its bioavailability-enhancing action [72,73]. According to the literature, a proper surfactant combination provides more efficient packing in the oil–water interfacial layer with increased rigidity of the interfacial film [74].

As revealed by Table 5, the terms (linear, quadric and interaction) of % S_mix_, % amplitude and sonication time showed significant influence on the ADS of the prepared NEs. The b values were greater for the linear terms of % S_mix_ and sonication time (*p* < 0.001), followed by the linear term of ultrasonic amplitude (*p* < 0.05). Figure 1 shows the response surface diagrams for ADS and illustrates the relationship between ADS and the two effects of two independent variables, while the third variable is kept at the central value. As seen from Figure 1A,B, during the nano-emulsification process, S_mix_ played a crucial role in droplet size determination. Initially, it was observed that ADS decreases noticeably with increasing S_mix_, followed by size enlargement with a further increase in S_mix_. At low % S_mix_, the emulsifier concentration in the system was inadequate oil droplet coverage, triggering the phenomenon of coalescence. By increasing the % S_mix_, the emulsifiers will adequately cover the surfaces of the formed droplets, decrease the interfacial tension, and stabilize the system. However, a further increase in % S_mix_ droplet size enlargement was observed, particularly when combined with high amplitude and sonication time [75]. High concentrations of emulsifiers ultimately increase the flow resistance in the emulsification process, which results in greater values of the apparent viscosity of the developed NEs. The increased viscosity, in turn, diminishes propagation of the ultrasonic waves created near the microtip surface and consequently reduces the shear stress induced by the acoustic cavitation. The overall ultrasound-assisted emulsification efficiency will be reduced with difficulty in the breakup of the oil–water interface with the formation of larger droplet sizes [40].

A specific amount of energy is necessary to induce cavitations for droplet size minimization in coarse emulsions. According to Figure 1A,C, low levels of % amplitude were efficient to generate the cavitation effect necessary for droplet breakup [75]. However, the excess energy supply at higher % amplitudes causes intense turbulence and promotes collisions between droplets, coalescence, with the formation of larger-sized droplets [40].

The impact of sonication time on the ADS of the sono-processed NE is displayed (Figure 1B,C). According to the results, ultrasonic processing for shorter times at different amplitudes was able to generate emulsions with low ADS values. However, this effect is reversed by extending the sonication time. The heat generated with time, particularly at a higher % amplitude, resulted in ADS enlargement of the acoustically formed NE. Moreover, high ultrasonic amplitude or longer irradiation affects the emulsifying properties of non-ionic CR emulsifiers and consequently leads to an induced droplet aggregation [29].

#### 3.2.2. PDI

The PDI values of the NE preparations are shown in Table 1, whereas Table 5 summarizes the results of the regression analysis of the generated models. Figure 2 depicts the links between the PDI and the effects of two independent variables, with the third variable remaining constant at the center point. As per Table 5, the *p*-values showed all terms (linear, quadric and interaction) of independent variables significantly affect the PDI of the generated NE. Higher b values were observed for the linear term of ultrasonic amplitude (*p* < 0.001), followed by linear terms of % S_mix_ (*p* < 0.01) and sonication time (*p* < 0.05). Initially, the PDI readings decrease by increasing emulsifier concentrations, % S_mix_, (Figure 2A,B). However, when the emulsifier concentration is increased (>10%), the droplet size became more heterogeneous, as reflected by the higher PDI values. The high surfactant concentrations plausibly exceeded the level at which a very viscous liquid around the droplet phase was generated, making spontaneous breakdown of the oil–water interface more difficult [76]. Similarly, the excess amount of energy causes intense turbulence and collisions among oil droplets resulting in a more pronounced drop–drop coalescence and larger PDI values (Figure 2A,C). Moreover, the observed PDI values were higher with prolonged sonication time (Figure 2B,C). Here, the used emulsifiers could be thermally deteriorated resulting in aggregating neighboring droplets and increasing PDI values [40].

#### 3.2.3. Optimization of Responses

**Figure 3:**
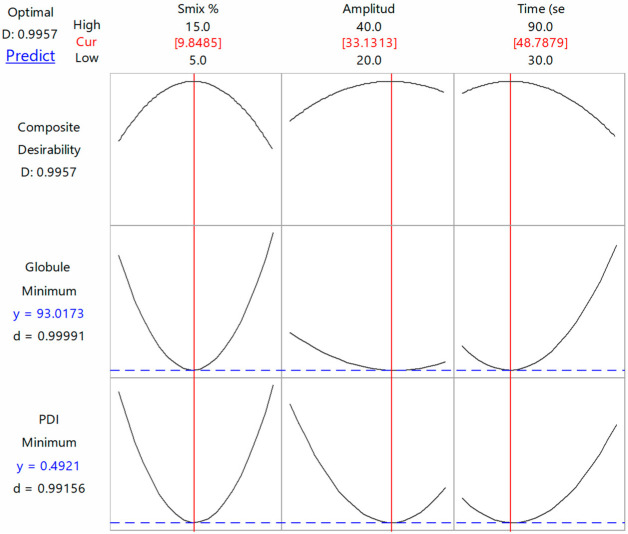
Optimization Box–Behnken design plot of ADS and PDI. Red line designates the present factor settings. Blue line designates the response to the present settings. Composite desirability value (D) approaching one indicates the settings seem to achieve satisfactory responses for all responses. The individual desirability value (d) indicates the value near to one thereby optimizing the settings for each individual response. The upper middle numbers (red) indicate the current optimum settings.

**Figure 4:**
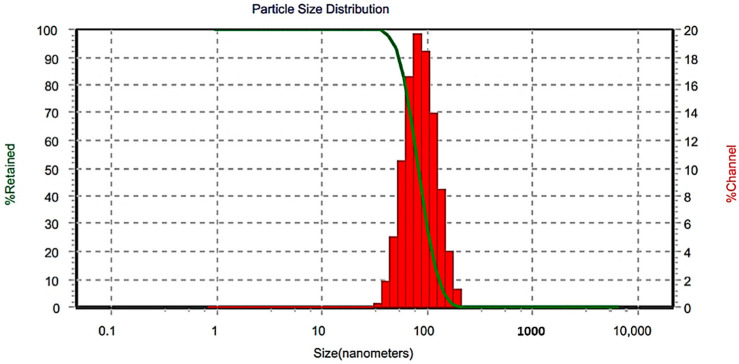
Size distribution of the optimized formulation.

The BBD optimization approach was utilized to discover the best combination of the tested processing variables to achieve the favorite responses. Here, the TAD-loaded NE is considered optimal when the optimization criteria resulted in the lowest ADS and narrowest PDI. The combined values; % S_mix_ = 9.85; ultrasonic amplitude % = 33; and sonication time = 49 s were found successful to fulfil requisites of the optimized tadalafil NE (OTN) with a composite desirability value (D) of 0.996 (Figure 3). A composite desirability value, D, near to one indicates that the settings seem to achieve favorable responses. Under these conditions, the generated response values for ADS and PDI were 93.02 nm and 0.49, respectively (Figure 4). The average response values of fresh NE samples were found to be 99.67 ± 7.55 nm for ADS and 0.45 ± 0.04 for PDI (Table 6). These findings suggested the adequacy of the related response regression equations linking the responses to independent variables (<15% of the percent bias).

### 3.3. Nano-Emulsion Stability

**Figure 5:**
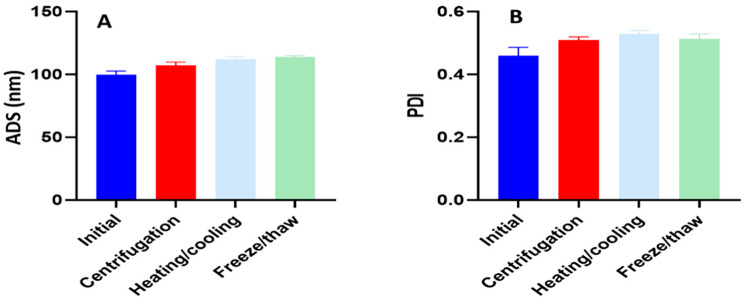
Nano-emulsion stability evaluation of the optimized tadalafil formulation; (**A**) changes in average droplet size (ADS) and (**B**) changes in polydispersity index (PDI).

A stable NE could endure a broad range of temperature changes and centrifugal stress without phase separation or drug precipitation [39]. According to the stability tests, the optimized formulation, OTN, did not demonstrate any precipitation or phase separation under the various investigated stress conditions. The changes in ADS and PDI after stability tests are represented in Figure 5. According to the results, the optimized formulation, OTN, did not demonstrate any obvious changes in size or PDI values. To assure the formation of uniform emulsions in conditions resembling the in vivo conditions, OTN was also evaluated for robustness in 0.1 N HCl and the phosphate buffer (pH 6.8) with variable dilution folds (10, 100, and 1000 times). No precipitation was observed, even after 24 h, with the continuing increase in dilution and change in dilution media with minimal changes in ADS and PDI values (Table 7). The Zeta-potential (ZP) value is a crucial parameter in the stability of nano-emulsions. A high ZP value promotes stability because a sufficiently significant charge can prevent droplet aggregation owing to electrostatic repulsion between the droplets [77]. The measured ZP (−23.4 ± 2.2 mV) is one of the plausible reasons for stability of OTN.

### 3.4. Oral Jelly Formulations

Sodium carboxymethyl cellulose (Sod. CMC) was selected as the gelling agent because of its inertness to cations in physiological fluids, pH-independent nature and processibility using cold water meaning an advantage for commercial manufacturing [48]. TAD-NEJ were successfully formulated using the optimized NE formulation (OTN). Table 8 summarizes the results of various parameters of the prepared jelly formulations. Physical assessment of the jellies is necessary to justify patient acceptance and compliance with the preparations. The jellies were mostly transparent with slightly liquid to thick consistencies. All of the NEJ showed non-sticky textures with acceptable color and odor. The TAD content was found in the range of 96.2 ± 0.8 to 98.7 ± 0.7%, which was in conformity with the pharmacopeial specification of 95–105%. The taste and stability of oral jellies are affected by the pH of the formulations [49]. Here, the pH values of the developed jellies ranged from 6.50 ± 0.04 to 6.83 ± 0.03, i.e., close to the neutral values required to enhance users’ acceptability [26]. The viscosities of NEJ were found between 3820 ± 98 and 10789 ± 277 cps and varied in accordance with the concentration of the gelling agent (Sod. CMC polymer). Satisfactory consistencies of jelly formulations were observed at gelling agent concentrations > 2%. Jellies may undergo syneresis or deswelling as a result of water separation, resulting in shrinkage and reduced quality [47]. In our study, formulations NEJ-1 and NEJ-2 showed syneresis at both 25 and 8 °C, whereas NEJ-3 showed syneresis only at 25 °C. Other formulations (NEJ 3-6) showed no signs of syneresis at the specified temperatures. These results suggest that syneresis was more evident at low concentrations of the gelling agent (<2% *w*/*w*). The findings of a taste-masking assessment of the NEJ formulations are presented in Table 8. The bitterness of TAD was substantially reduced by the incorporated additives, as reflected in the scores given by the volunteers. The decrease in bitterness might be attributed to the slow diffusion of TAD from the formulations to the human taste receptors by the gelling agent [49].

#### 3.4.1. DSC

**Figure 6:**
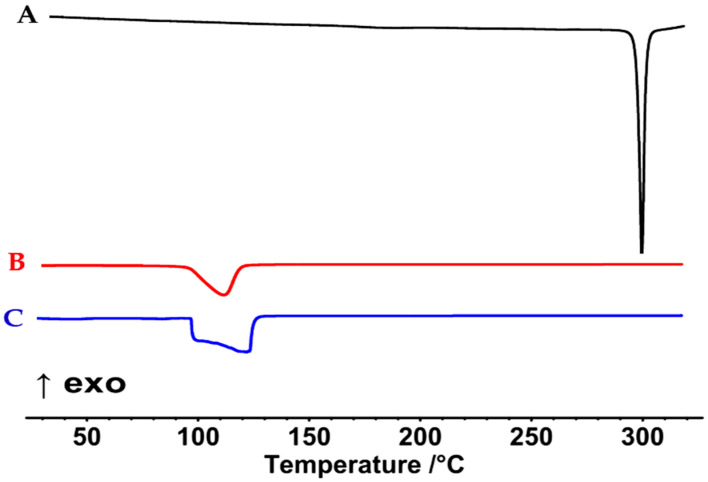
DSC thermograms of the unprocessed tadalafil (A), the nano-jelly formulation (B) and the corresponding blank formulation (C).

The DSC thermogram of unprocessed TAD shows a sharp endotherm at 305 °C that corresponds to the TAD melting point (Figure 6A). This was in close agreement with previously published data and suggests an orderly crystalline compound [78]. Nevertheless, as seen in Figure 6B, the TAD representative peak disappeared completely in the DSC thermogram of the optimized jelly formulation, NEJ-5. Apparently, TAD exists in a dissolved/amorphous state within the jelly matrix. Peaks at 110–140 °C in Figure 6B,C, are more likely linked to Na CMC [79,80].

#### 3.4.2. Reconstitution

**Figure 7:**
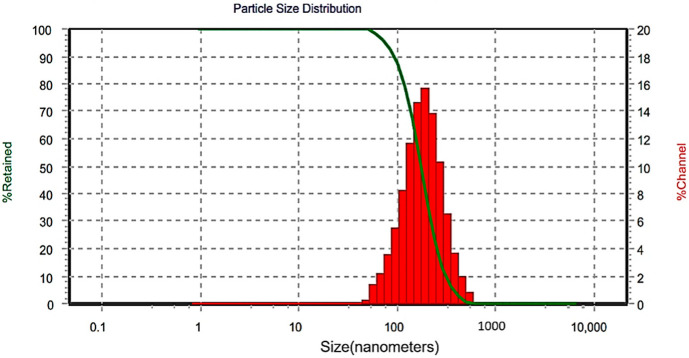
Size distribution of the nano-emulsion-based jelly formulation (NEJ-5) after reconstitution.

The result of NEJ-5 reconstitution is presented in Figure 7. The ADS and PDI of the reconstituted NE from NEJ-5 were 139.0 ± 15 nm and 0.56 ± 0.07, respectively. These values are nearly similar to the corresponding initial values of the original NE. This finding confirms the retaining of the nano-emulsification capability of the formulation.

#### 3.4.3. Dissolution

**Figure 8:**
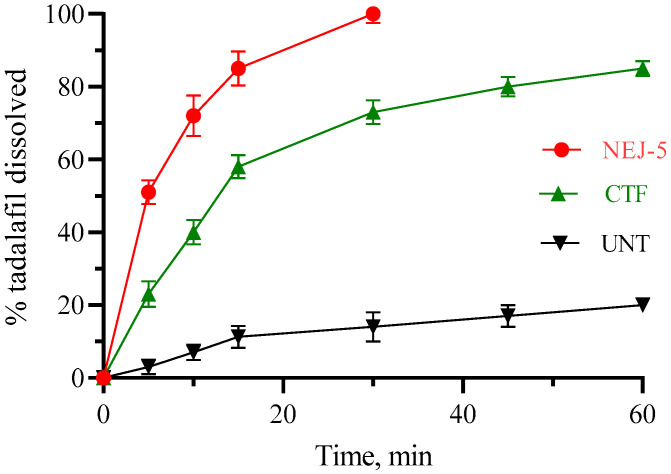
Dissolution of tadalafil from nano-emulsion-based jelly (NEJ-5), commercial tablet formulation (CTF) and unprocessed tadalafil powder (UNT).

Findings of the in vitro dissolution study of TAD from the nano-emulsion-based jelly (NEJ-5), the commercial tablet formulation (CTF) and the unprocessed tadalafil powder (UNT) are displayed in Figure 8. Generally, the steps of water diffusion, polymer chain relaxation, swelling, and jelly erosion are involved in drug dissolution from jelly formulations [47]. According to the results, the percentage of TAD dissolved after 10 min (%Q_10min_) was 72 ± 5.6, 40 ± 3.4, 7 ± 2.1, respectively. The dissolution of TAD from the NEJ-5 formulation reached 100% within 30 min. The higher dissolution rate from the NEJ-5 formulation is plausibly attributed to the spontaneous generation of drug-loaded nano-emulsified droplets with a large surface area, the drug’s molecular dispersion, and the wetting and high solubilization capabilities of the surfactant and cosurfactant mixes [81,82].

#### 3.4.4. Stability

The maintenance of jelly characteristics such as viscosity, color, clarity, taste, and odor throughout its shelf-life is crucial for physically stable oral jellies. The stability of NEJ-5 was assessed by comparing the characteristics of the samples before and after the study period (Table 9). Statistical analysis revealed no significant changes in physical characteristics and drug content of the optimized NEJ-5 formulation of TAD during stability studies (*p* < 0.05). The viscosity values, %Q_10min_, ADS, PDI, pH and drug content were not significantly altered during the stability testing of the formulation. Accordingly, stability data confirmed the maintenance of the quality of the NEJ-5 product over the period of the study.

#### 3.4.5. Bioavailability

**Figure 9:**
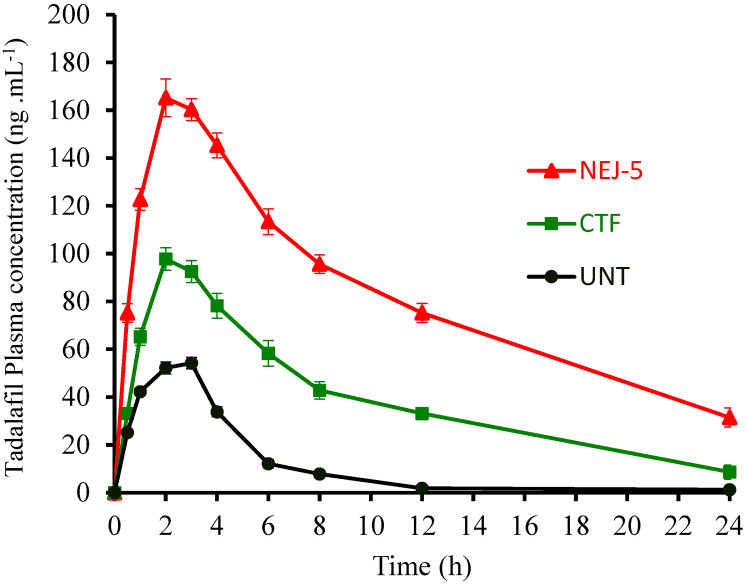
In vivo assessment of tadalafil formulations: nano-emulsified jelly (NEJ-5), commercial tablet formulation (CTF) and unprocessed tadalafil (UNT).

The results of the bioavailability study performed on TAD preparations are displayed in Figure 9, while the pharmacokinetic parameters are listed in Table 10. In consistency with the in vitro dissolution study, the NEJ-5 formulation demonstrated a greater absorption profile than the UNT, which is plausibly attributed to the poor dissolution of TAD in its raw form. The value of C_max_ of the optimized NEJ-5 (165.2± 7.9, ng·mL^−1^) was significantly higher compared to CTF and UNT (*p* < 0.05). The mean residence time (MRT) values for NEJ-5, CTF and UNT were found to be 9.3 ± 0.8 h, 6.87 ± 0.7 h and 3.55 ± 0.53, respectively (*p* < 0.05). The time of peak plasma concentration (t_max_) for NEJ-5 (1.95 ± 0.2 h) was shorter than UNT (*p* > 0.05). The bioavailability indicator, AUC_0–24h_ (1685 ± 77 ng·h·mL^−1^), was also significantly higher than CFT (933.0 ± 49 ng·h·mL^−1^) and UNT (259.9 ± 18 ng·h·mL^−1^). The calculated relative bioavailability of NEJ-5 in comparison to the commercial formulation was 180%. The generation of drug-loaded nano-sized droplets with large effective surfaces and increased contact time led to the rapid absorption of TAD from NEJ-5. In addition, the extended residence time of NEJ-5 in the gastrointestinal tract surface due to the adhesiveness properties of sodium CMC allows the drug particles to adhere to the absorption sites for a longer period of time, increasing the absorption extent and drug plasma level [83]. One more reason for the enhanced bioavailability is the avoidance of recognition by *p*-gp or the putative suppressive effects of the added excipients such as Capryol 90 and Transcutol HP in NEJ-5 [84].

## 4. Conclusions

In the current study, sono-assisted nano-emulsions were generated. The impact of the emulsifier concentration, ultrasonication amplitude level (%) and ultrasonication duration was investigated and optimized using the Box–Behnken design approach. The ultrasonication amplitude level, emulsifier concentration and sonication time all showed an inverse effect on droplet characteristics. Nano-emulsion-based jellies (NEJ) were formulated using the optimized nano-emulsion. The results from characterizing the developed NEJ showed tadalafil dissolution rate and absorption enhancement. Effectively, the developed NEJ is advantageous to deliver tadalafil in a palatable easily administered dosage form, with immediate-release characteristics and enhanced bioavailability. Nevertheless, a toxicity study is further required to ensure the safety of the developed NEJ formulation by oral route.

## Figures and Tables

**Table 1:** The Box–Behnken design showing variables of the nano-emulsification procedures.

Factor	Levels		
Independent Variables	Low	Intermediate	High
X_1_ (% S_mix_)	−1	0	+1
X_2_ (% Amplitude)	−1	0	+1
X_3_ (Sonication time)	−1	0	+1
Translation of coded levels in actual units			
Coded levels	Low (−1)	Intermediate (0)	High (+1)
X_1_ (% S_mix_)	5	10	15
X_2_ (% Amplitude)	20	30	40
X_3_:(Sonication time, sec)	30	60	90
Dependent variables	Target		
Y_1_ = ADS: Average droplet size (nm)	Minimize		
Y_2_ = PDI: Polydispersity index	Minimize		

**Table 2:** Results of randomized experiments of the Box–Behnken design.

Run	X_1_	X_2_	X_3_	Y_1_	Y_2_
1	0	−1	1	182 ± 11.4	1.29 ± 0.09
2	−1	−1	0	178 ± 18.3	1.91 ± 0.08
3	1	0	1	289 ± 30.6	1.40 ± 0.1
4	1	−1	0	245 ± 19.8	1.50 ± 0.17
5	0	1	1	205 ± 11.9	1.31 ± 0.13
6	1	0	−1	216 ± 16.7	1.45 ±0.09
7	−1	0	−1	197 ± 11.6	1.25 ±0.11
8	−1	0	1	244 ± 20.5	1.78 ± 0.18
9	0	0	0	105 ± 11.8	0.57 ± 0.04
10	1	1	0	196 ± 15.8	1.40 ±0.03
11	0	0	0	93 ± 9.8	0.55 ± 0.72
12	0	1	−1	110 ± 7.9	0.72 ± 0.09
13	0	−1	−1	148 ± 12.8	1.30 ± 0.08
14	0	0	0	103 ± 11.7	0.48 ± 0.06
15	−1	1	0	200 ± 18.7	1.38 ± 0.15

**Table 3:** Tadalafil jelly formulations.

Ingredient (g)	Formulations					
	NEJ-1	NEJ-2	NEJ-3	NEJ-4	NEJ-5	NEJ-6
Tadalafil	0.05	0.05	0.05	0.05	0.05	0.05
Cinnamon essential oil	0.3	0.3	0.3	0.3	0.3	0.3
Capryol^TM^ 90	6.7	6.7	6.7	6.7	6.7	6.7
Cremophor RH 40	8	8	8	8	8	8
Transcutol HP	2	2	2	2	2	2
Sodium CMC	1	1.5	2	2.5	3	3.5
Stevia	1	1	1	1	1	1
Amaranth	0.1	0.1	0.1	0.1	0.1	0.1
Strawberry flavor	0.5	0.5	0.5	0.5	0.5	0.5
Methyl paraben	0.2	0.2	0.2	0.2	0.2	0.2
Distilled water to	100	100	100	100	100	100

**Table 4:** Findings of preformulation studies.

Solubility Study	
Vehicle	Solubility (mg·g^−1^)
Cinnamon essential oil	54.60 ± 3.2
Capryol^TM^ 90	9.12 ± 1.7
Cremophor^®^ RH	26.60 ± 3.1
Transcutol^®^ HP	23.60 ± 2.7
Distilled water	0.01 ± 0.009
HLB of the system	
% Cremophor^®^ RH 40	71.4
% Transcutol^®^ HP	28.6
HLB of S_mix_	11.922
Transparency (%)	97.5
Visual aspects	Homogeneity

**Table 5:** Findings of regression analysis.

Variable	ADS (nm)		PDI	
	b	*p*-Value	b	*p*-Value
Intercept	709	<0.01	8.715	<0.01
Linear				
X_1_	−60.64	0.000	−0.5524	0.009
X_2_	−8.77	0.025	−0.2761	0.000
X_3_	−7.099	0.000	−0.03719	0.001
Quadric				
$X_{1}^{2}$	3.5933	0.000	0.02658	0.000
$X_{2}^{2}$	0.1458	0.020	0.003496	0.000
$X_{3}^{2}$	0.0515	0.000	0.003496	0.000
Interaction				
${\;X}_{1}X_{2}$	−0.3550	0.001	0.002150	0.007
${\;X}_{1}X_{3}$	0.0433	0.040	−0.000967	0.002
${\;X}_{2}X_{3}$	0.05083	0.001	0.000500	0.002
Model statistics				
R^2^	99.77%		99.56%	
Adjusted R^2^	99.35%		98.77%	
Predicted R^2^	98.67%		95.26%	
Lack of fit F-value	0.23		1.1	
Lack of fit *p*-value	0.871		0.508	

**Table 6:** Findings of the tadalafil nano-emulsion optimization.

Predicted Values of Independent Parameters	Responses	Predicted Values	Experimental Findings	* Percent Bias
X_1_ = 9.85%	Y_1_ = Average droplet size (ADS)	93.02	99.67 ± 7.55	−6.9
X_2_ = 33%	Y_2_ = Polydispersity index (PDI)	0.49	0.45 ± 0.04	8.16
X_3_ = 49 s				

* % bias = (predicted value-observed value)/(predicted value) × 100.

**Table 7:** Effect of dilution on the optimized tadalafil NE (OTN) characteristics.

Formulation	Dilution Fold	0.1 N HCl		Phosphate Buffer (pH 6.8)	
		ADS	PDI	ADS	PDI
OTN	10	99 ± 9.4	0. 48± 0.03	102 ± 9.7	0.50 ± 0.08
	100	103± 12.9	0. 47± 0.10	105 ± 8.6	0.52 ± 0.07
	1000	108 ± 9.3	0. 49± 0.05	113 ± 15.3	0.53 ± 0.11

**Table 8:** Characteristics of tadalafil NE jellies (TAD-NEJ).

Code	Consistency	Texture	Transparency Score	Drug Content	Viscosity (cps)	pH	Syneresis		Taste-Masking Score
							25 °C	8 °C	
NEJ-1	Slightly liquid	Non-sticky	4	98.7 ± 0.7	695 ± 89	6.50 ± 0.04	Yes	Yes	2
NEJ-2	Slightly liquid	Non-sticky	4	96.4 ± 0.9	1832 ± 144	6.55 ± 0.05	Yes	Yes	2
NEJ-3	Slightly liquid	Non-sticky	4	97.2 ± 0.8	3211 ± 188	6.67 ± 0.07	Yes	No	2
NEJ-4	Acceptable	Non-sticky	4	96.9 ± 0.7	6560 ± 276	6.78 ± 0.06	No	No	1
NEJ-5	Acceptable	Non-sticky	4	96.5 ± 0.3	7765 ± 211	6.81 ± 0.04	No	No	1
NEJ-6	Slightly thick	Non-sticky	3	96.2 ± 0.8	9865 ± 233	6.83 ± 0.03	No	No	1

**Table 9:** Results of stability studies on the optimized formulation after storage for 3 months.

Temperature (°C)	Viscosity (cps)	pH	Drug Content (% *w*/*w*)	ADS	PDI	%Q_10min_
Fresh samples	9234 ± 244	6.81 ± 0.04	96.5 ± 0.3	139.0 ± 15	0.56 ± 0.07	72 ± 5.6
After 3 months	9789 ± 169	6.82 ± 0.05	96.2 ± 0.67	152 ± 13	0.58 ± 0.09	69 ± 6.1

**Table 10:** Pharmacokinetic parameters of tadalafil in rats after oral administration.

PK Parameter	NEJ-5	CFT	UNT
C_max_, ng·mL^−1^	165.2± 7.9	97.8 ± 4.3	52.9 ± 4.0
t_max_ (h)	1.95 ± 0.2	1.90 ± 0.3	2.2 ± 0.3
AUC_0–∞_ (ng·h·mL^−1^)	1685 ± 77	933.0 ± 49	259.9 ± 18
MRT (h)	9.3 ± 0.8	6.87 ± 0.7	3.55 ± 0.53

PK: Pharmacokinetic parameter; NEJ-5: Nano-emulsified jelly; CTF: commercial tablet formulation, UNT: unprocessed tadalafil.

## Data Availability

Data sharing is not applicable to this article.
